# Prevalence and risk factors for lameness in dairy cattle on selected farms located in Dessie and Kombolcha, Northeast Ethiopia

**DOI:** 10.3389/fvets.2025.1456527

**Published:** 2025-04-28

**Authors:** Hasen Ahimed Mekonin, Abadi Amare Reda, Alula Alemayehu Assen, Awol Mohammed Assen

**Affiliations:** ^1^School of Veterinary Medicine, Wollo University, Dessie, Ethiopia; ^2^Animal Genetics and Breeding Unit (AGBU), NSW Department of Primary Industries and Regional Development and University of New England, Armidale, NSW, Australia; ^3^International Livestock Research Institute, Addis Ababa, Ethiopia; ^4^Animal Science, School of Environmental and Rural Science, University of New England, Armidale, NSW, Australia

**Keywords:** dairy cows, lameness, management, animal- and herd-level risk factors, prevalence, prevention

## Abstract

**Background:**

Lameness in dairy cattle has continued to be a significant burden for farmers in modern dairy production due to its impact on animal welfare and productivity. A cross-sectional study was conducted from May 2022 to February 2023 to estimate the prevalence and identify associated risk factors of lameness in 433 dairy cows across 37 selected farms located in Dessie and Kombolcha, Northeast Ethiopia.

**Methods:**

The selected animals were examined for lameness using a five-point visual locomotion scoring technique during daily outdoor access on a solid walking surface in the designated refreshment areas. Cows with a lameness score of > 2 were considered clinically lame. The overall prevalence of lameness was defined as the total number of clinically lame animals divided by the total number of animals examined. The herd-level prevalence was calculated as the total number of positive herds divided by the total number of herds sampled. After variable screening using univariable analysis, separate multivariable mixed-effects logistic regression models that included farm as a random effect were fitted to identify risk factors for lameness at both the animal and herd levels.

**Results:**

The overall prevalence of lameness was 5.77% (95%CI = 3.57–7.98%). The herd-level lameness prevalence was 32.4% (95%CI = 18.0–49.8%), while the average within-herd lameness prevalence was 5.20% (95%CI = 2.46–7.95%, range = 0.00–25.0%). The animal- and herd-level risk factors included in the final multivariable mixed-effects model were age, body condition score, milking status, and farm history of lameness. Among these, only milking status and lameness history were significant in the final model. The odds of being lame were higher in the cows in the middle (OR = 10.8, 95%CI = 1.37–84.8, *p* = 0.024) and late (OR = 11.1, 95%CI = 1.38–88.8, *p* = 0.024) stages of lactation. Furthermore, the animals on farms with a history of lameness (OR = 10.0, 95%CI = 2.87–37.4, *p* = 0.001) were more likely to be clinically lame.

**Conclusion:**

Lameness was strongly associated with the middle and late stages of lactation, particularly on farms with a previous history of lameness. Therefore, farmers should regularly monitor and maintain cows’ lactation status and increase their awareness about lameness on farms to help reduce its occurrence.

## 1 Introduction

Lameness is a clinical sign of any painful/uncomfortable condition affecting the locomotory system of dairy cattle, characterized by abnormal movement and posture (1, 2). It continues to be a significant burden for farmers in the modern dairy industry for several reasons. First, after mastitis and infertility, lameness is one of the leading conditions affecting the health and productivity of dairy herds globally (2–4). Second, lameness is a major animal welfare issue of societal concern, as it results in pain and restricts the free movement of dairy cows (5, 6). Third, lameness causes financial losses for dairy farmers due to reduced weight gain and milk yield, increased medication costs, culling of lame cows (especially at an early age), and infertility, among other factors (1, 7–10). Fourth, lameness affects the sustainability of dairy farming (11, 12) as it increases the likelihood of culling lame cows with foot lesions, particularly among lactating cows and primiparous heifers (13). Finally, it leads to the frequent use of antibiotics for the treatment of lame animals on dairy farms (14), which contributes to the emergence of antimicrobial resistance (15).

The causes of lameness in dairy cattle are varied and can originate from several factors. A previous review (3) pointed out that hoof/claw lesions are the predominant cause of lameness, which can be classified as infectious or non-infectious. Non-infectious claw lesions include white line disease, sole lesions/ulcers, sole hemorrhage, and interdigital hyperplasia, while infectious lesions include digital dermatitis, heel erosion, and foot rot (16, 17). The prevalence of lameness is associated with numerous factors related to the animal or the herd/environment. For example, animal-level factors include the presence of claw overgrowth, breed, age, body condition score, milk production, herd size, lactation stage, and parity (2). The herd-level/environmental factors are mainly associated with the housing system, including floor type, stall design, bedding thickness, type of bedding materials, and access to pasture. Other management factors, such as stall hygiene and the frequency of hoof trimming, have also been associated with the prevalence of lameness in dairy cows (18). Therefore, monitoring the prevalence of lameness in animals and herds, quickly detecting lame cows, and implementing effective therapeutic measures are essential for preventing lameness on dairy farms (19, 20). As a result, the duration and prevalence of lameness can be reduced, thereby improving the production and welfare of cows (21, 22).

According to a more recent review (23), the average global prevalence of lameness (i.e., score ≥3) was 22.8%, with a median of 22.0% and a range of 5.1 to 45%. In addition, the within-herd lameness prevalence ranged from 0 to 88%. However, the average prevalence of severely lame cows (i.e., score ≥4) was 7.0%, with a range of 1.8 to 21.2%, and the within-herd prevalence ranged from 0 to 65%. Furthermore, the prevalence of hock injuries was high, with within-herd estimates ranging from 12 to 81% of cows affected globally (18). Studies conducted in Ethiopia reported cow-level prevalence of lameness ranging from 2 to 25.7%, with herd-level prevalence ranging from 47 to 55% (24). The most common animal-related risk factors associated with the prevalence of the disease were stage of pregnancy, parity, and milking status. In addition, the practice of hoof trimming and floor type were among the farm-level risk factors associated with the occurrence of lameness in Ethiopia (25–27).

According to a previous study (28), milk yield significantly decreases immediately after the onset of lameness. The economic impact of lameness includes a considerable reduction in milk yield and increased treatment costs. On average, the financial loss due to reduced milk yield and increased treatment costs for a clinically lame lactating cow in Wolaita Sodo was estimated at 7.33 USD. This cost is significant for a typical smallholder dairy farmer in developing countries, particularly in sub-Saharan Africa, where their livelihood relies on dairy production. As lameness adds an additional economic burden for smallholder dairy farmers, measuring its prevalence and identifying the potential risk factors is necessary. This offers opportunities for designing early lameness detection and prevention strategies. Quantifying animal- and herd-level prevalence of lameness and identifying the associated risk factors are crucial for developing measures to improve the health and welfare of cows (23, 29). Despite the significant impact of lameness on the health and welfare of dairy cattle, as well as the increased economic burden on farmers in Ethiopia, there is a lack of information on the magnitude and associated risk factors of lameness, specifically in the South Wollo zone. Therefore, this study aimed to determine the prevalence and associated risk factors of lameness in dairy cattle on selected farms located in Dessie and Kombolcha, Northeast Ethiopia.

## 2 Materials and methods

### 2.1 Description of the study area

**Figure 1:**
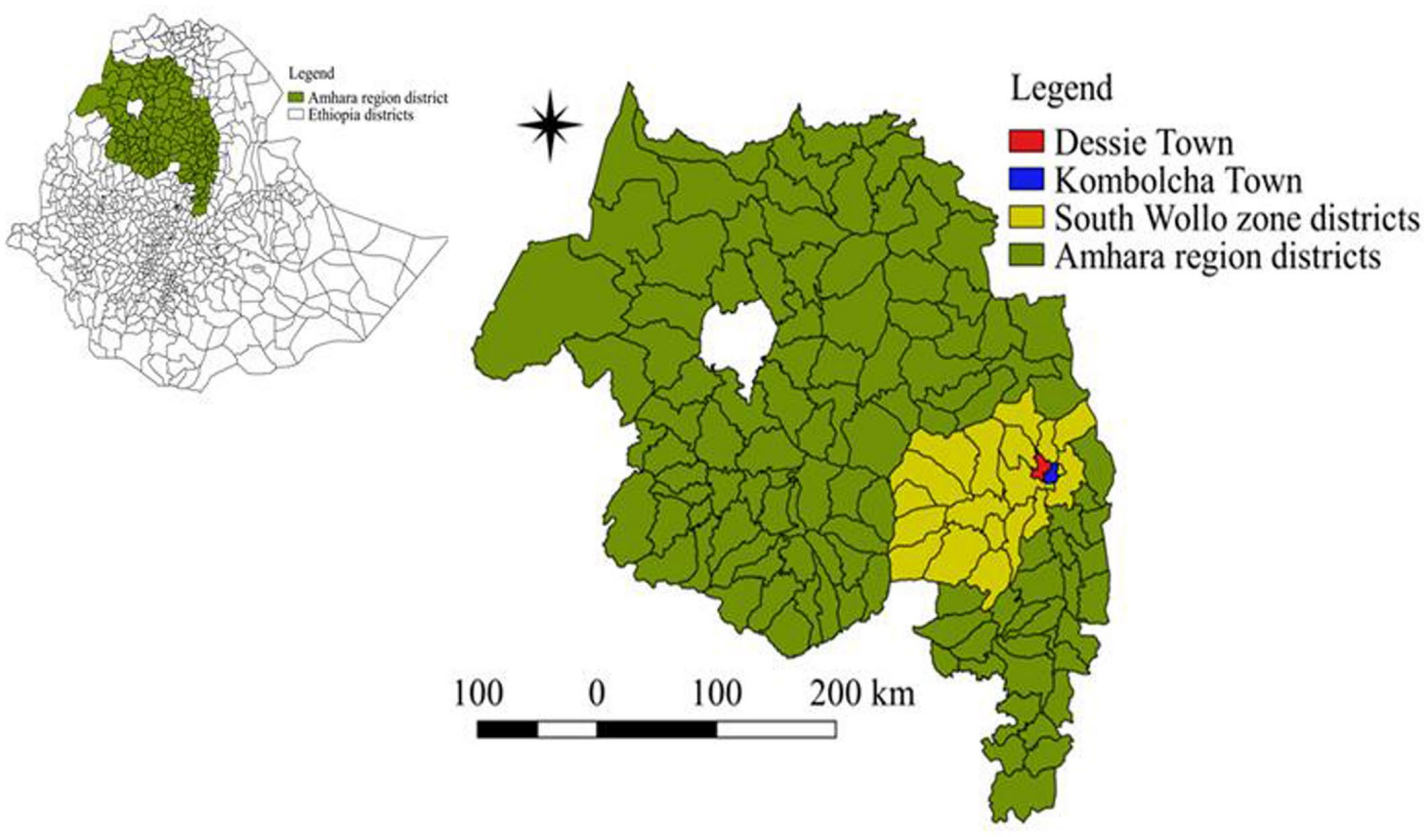
Map of the study areas (Dessie and Kombolcha towns) located in the South Wollo zone, Amhara region, northeast Ethiopia.

A study was conducted to determine the prevalence of dairy cattle lameness and associated animal- and herd-level risk factors from May 2022 to February 2023. The study was carried out on commercial and smallholder dairy farms in Dessie and Kombolcha towns, located in the South Wollo zone of the Amhara region, Northeast Ethiopia (Figure 1). Dessie and Kombolcha towns are key milk-shed areas. These towns were selected because the regional administration promotes dairy crossbreeding through the use of artificial insemination and by distributing heifers and pregnant crossbred Holstein cows. These areas are favorable for dairy production due to the large number of dairy cattle and a high human population with significant demand for milk.

### 2.2 Study design, farm selection criteria, and sampling technique

A cross-sectional study was conducted to determine the prevalence and to identify the associated risk factors of dairy cattle lameness across 37 dairy farms. A list of 200 dairy farms was obtained from Livestock and Fishery Development Office in Dessie (*N* = 53) and Kombolcha (*N* = 147). From this list, 100 farms (Dessie, *N* = 26; Kombolcha, *N* = 74 farms) were purposively selected based on predefined inclusion criteria. The willingness of farm owners to participate in the study, farm accessibility, and the availability of recorded information were used as inclusion criteria. Moreover, only farms with five or more cows or heifers (at least 1-year-old) were included. Individual farms were considered as clusters. After applying the selection criteria, 37 farms were selected for the study using a cluster random sampling technique. Once the farms (clusters) were selected, all cows and heifers within the selected farms were included in the study and examined for lameness. These farms were visited once, and during each farm visit, the occurrence of lameness was assessed using the visual locomotion score (VLS) technique for all study animals. When the animals were diagnosed as lameness-positive, the causes of lameness were also identified with the help of farm veterinarians. Generally, the majority of the selected farms had visiting/on-call veterinarians who monitored the health aspects and looked after cows at the time of giving birth. In addition, an interview using a structured questionnaire was conducted with the farmers to collect information regarding each enrolled cow and herd-level data related to risk factors for dairy cattle lameness during the individual farm visits (Supplementary material 1). The gait scoring method was used to detect lameness and was conducted during daily outdoor access on a hard walking surface in the refreshment areas.

### 2.3 Lameness assessment

The selected animals were examined for lameness using the VLS method. All study animals were permitted to be mobile and were examined for any signs of abnormal gait or visual locomotion as part of the clinical diagnosis of foot and leg lameness in cattle (30). The cows with an apparent abnormal gait were clinically examined for any signs of pain or discomfort, and the examination focused on identifying the anatomical part affected, the type of lesions, their cause, and the extent of lesions. The observed locomotion was scored using a 5-point rating system, where 1 = normal, 2 = mildly lame, 3 = moderately lame, 4 = lame, and 5 = severely lame (31, 32). Accordingly, the animals with a VLS of >2 were considered lame.

### 2.4 Sample size determination

The sample size was determined using the sample size determination equation (Equation 1) described in a previous study (33), with a 95% confidence interval and a 5% desired precision. Since there was no previous study conducted in this area, an expected prevalence of 9.2% for lameness in Debre Berhan (34) was used to determine the number of animals for this study. The formula used is described as follows:


(1) $$n=\frac{\left({\left(1.96\right)}^{2}\;\left[\mathrm{Pexp}\;\left(1-\mathrm{Pexp}\right)\right]\right)}{d^{2}}$$

Where *n* = sample size, *Pexp* = expected prevalence, and *d* = desired absolute precision. Accordingly, (1.96^2^ × 0.092 (1–0.092))/0.0025 = 128. The calculated sample size was 128 dairy cows and heifers. However, to improve the precision of our estimate, a total of 433 animals were included in this study. Finally, the study was conducted on 433 crossbred dairy cows and heifers from 37 farms.

### 2.5 Description of the study variables

The prevalence of dairy cattle lameness (at the animal and herd levels) with a binary outcome (1 = lame, 0 = normal) was the dependent variable in this study. Therefore, the proportion of clinically and severely lame cows and heifers on the day of the farm visit was calculated for each farm/herd. The animal-level hypothesized risk factors such as age, parity, pregnancy status, milking status, and body condition score (BCS) were explanatory variables. In addition, herd-level risk factors such as herd size, management system, type of stall, floor type, use of bedding material, exercise area, frequency of barn cleaning, animal washing, and hoof trimming practices were also explanatory variables.

The age of animals was determined based on the dental formula described in a previous study (35). After determining the ages of the animals, they were classified into three age groups: 1–2.5 years, 2.6–6 years, and over 6 years. During the study period, the animals’ body condition was classified as either good or poor, following the body condition scoring method recommended in a previous study (36). An animal was given a good body condition score if there was noticeable fat covering the areas on either side of the head and tail, making them soft to the touch, and if the spinous process could only be felt with very firm pressure. A poor body condition score was assigned when each spinous process could be easily identified and felt, either round or sharp to the touch. Other variable categories included animal class (heifers and cows) and parity (heifers, primiparous, and multiparous). The milking status of the cows was categorized into four levels: dry, early lactation [14–100 days postpartum], middle lactation [100–200 days postpartum], and late lactation [200–305 days postpartum]. Moreover, according to the stages of pregnancy, the cows were classified into four groups: non-pregnant, first trimester, second trimester, and third trimester. The management systems of the selected farms was classified into two levels: intensive [high-input, high-output system where livestock are managed under controlled conditions] and semi-intensive [moderate-input, moderate-output system that combines features of intensive and extensive systems]. The types of barn/housing systems were classified as follows: stall barns, in which animals are kept in individual cubicles with designated space, and loose barns, in which animals move freely within an open area. In addition, the availability of an exercise area (yes or no), use of bedding material (yes or no), floor type (soil and concrete), frequency of barn cleaning per day (once, twice, and three times), animal washing practice (yes or no), and hoof trimming practice (yes or no) were coded. Herd size was also classified into three categories based on the number of animals: small (5–10 animals), medium (11–24 animals), and large (more than 24 animals).

### 2.6 Data management and analysis

All collected data were stored in a Microsoft Excel spreadsheet, edited, coded, and summarized using descriptive statistics. Statistical analyses were performed using R version 4.4.2 (37). The individual farm was considered the experimental unit. Several parameters (variables) were not analyzed due to a lack of variability or a large proportion of missing data. These included age at first calving, milk yield, calving interval, days open, breed (crossbred local Zebu or *Bos indicus* breed with Holstein), and measures taken for lameness management. Continuous variables, such as age, herd size, milk yield, and age at first calving (AFC), were also categorized into quartiles.

The prevalence of lameness and the 95% confidence interval for the animal- and herd-level factors were estimated using the “epiR” package (38). The overall prevalence of lameness was calculated as the proportion of clinically lame animals to the total number of animals examined, multiplied by 100. The herd-level prevalence of lameness was calculated as the total number of positive herds divided by the total number of herds sampled. The within-herd prevalence was estimated as the average prevalence (i.e., the number of positive animals divided by the total number of animals sampled) for each herd. The herd-level lameness prevalence across 37 dairy herds was plotted using bar charts in Microsoft Excel.

Potential predictors were first visualized graphically to gain insights into the distributions and to understand correlations between predictors before analysis. Lameness outcomes were collected for individual animals, which are nested within farms, and farms are, in turn, nested within districts. The herd size and the prevalence of clinical lameness differed between the farms, so the baseline probability of lameness varied. Therefore, we used a mixed-effects logistic regression model with animal-level predictors as fixed effects and a random intercept by farm/herd. Farm was fitted as a random effect because of the significant variation in clinical lameness between the herds. A generalized linear mixed model with a complementary log–log link function was used to assess the association between the potential predictors and the lameness outcome using the lme4 package (39). Univariable mixed-effects logistic regression analysis was carried out for each explanatory variable individually to determine associations with the outcome variable, which is lameness prevalence. Farm or herd was included as a random effect to account for clustering. Variables with a *p*-value of ≤ 0.20 in the univariable analysis were included in a multivariable mixed-effects logistic regression model (40). Multicollinearity among the potential predictor variables was checked using the “performance” package (41) before establishing the final model. All predictors with a correlation of <0.6 were included in the final model (42). A separate multivariable mixed-effects logistic regression model for both animal- and herd-level predictors was constructed (43) using 10 adaptive Gauss–Hermite quadrature points. For risk variables at the animal and herd levels, farm and municipality were fitted as random effects, respectively. A binomial distribution with a complementary log–log link function was used to account for the low frequency of lameness observed across categories of explanatory variables. The final model was constructed using a backward elimination process.

The confounding effects of the predictors were assessed by examining the extent of the changes in the estimates (coefficients) of the categories of a predictor or the remaining explanatory variables after eliminating any variables. A variable was considered a confounder when the estimates of its categories changed by more than 30% (42). When these predictors were biologically significant and potential confounders, they were retained in the final multivariable model. The models’ goodness-of-fit and performance, including the Akaike information criterion (AIC), were evaluated using the “performance” package in R (41). Model diagnostics were performed by plotting the residuals to check for normality and homogeneity of variance using the “performance” package. The variables included in the final model for animal-level risk factors were age (1–2.5 years, 2.6–6 years, and >6 years), BCS (good and poor), and milking status (early, middle, late, and dry). Parity was omitted from the final model because the model failed to converge when parity was added. The herd-level risk factors were barn type (stall and loose barn) and farm lameness history (yes or no). In the final multivariable animal- and herd-level factor analysis, age was retained in the final model as it was both a confounder and biologically relevant factor. Only variables with *p*-values of <0.05 were retained in the final multivariable mixed-effects logistic model. The study considered a 95% confidence interval and a *p*-value of less than 0.05.

## 3 Results

A total of 433 animals from 37 farms located in Dessie (*n* = 168) and Kombolcha (*n* = 265) were included in this study. The median and mean herd size per farm were 17 and 19.6 (range = 5–49 animals), respectively. Table 1 presents descriptive statistics for all categorical and continuous variables in the dataset.

**Table 1:** Descriptive statistics of continuous variables of the dataset.

Variables	NA’s	*N*	First quartile	Median	Mean	SD	Third quartile	Range
Age (year)	0.00	433	2.50	4.50	4.667	2.63	6.000	1.0–15.0
Milk yield (L/cow/day)	155	278	7.00	10.0	11.42	5.81	15.00	2.0–40.0
AFC (years)	139	294	2.00	2.00	2.061	0.25	2.000	1.6–4.00
CI (years)	265	168	1.00	1.00	1.055	0.21	1.000	1.0–2.00
Days open (days)	221	212	60.0	60.0	73.68	49.0	60.00	40–400

AFC, age at first calving; CI, calving interval; NA’s, missing values; *N*, number of animals excluding NA’s; SD, standard deviation; First quartile, top 25%; Third quartile, top 75%.

### 3.1 Prevalence of dairy cattle lameness

The frequency of lameness in dairy cattle across animal-level factors is provided in Table 2. The visual locomotion score (VLS > 2) revealed that 25 out of 433 animals were lame, resulting in a cow-level prevalence of 5.77% (95% CI = 3.57–7.98%). According to Table 2, the prevalence of lameness was higher in multiparous cows, 9.17% (95% CI = 5.69–13.8%); middle-stage lactation cows, 15.2% (95% CI = 8.58–24.2%); and cows older than 6 years, 12.3% (95% CI = 7.54–18.5%).

**Table 2:** Prevalence of lameness and mixed logistic regression analysis of univariable associations expressed as odds ratios for animal-level factors in 433 cows across 37 South Wollo dairy herds in Dessie and Kombolcha.

Factors	Categories	*N*	Prevalence (95% CI)	Odds ratio	95% CI	*p*-value
Age*	1–2.5 years	131	0.76 (0.02–4.18)	Base		0.001^a^
	2.6–6 years	147	3.40 (1.11–7.76)	4.56	0.53–39.3	0.168
	>6 years	155	12.3 (7.54–18.5)	19.6	2.59–147.8	0.004
Parity	No parity	155	0.65 (0.02–3.54)	Base		0.025^a^
	Primiparous	60	6.67 (1.85–16.2)	15.3	1.63–143.4	0.017
	Multiparous	218	9.17 (5.69–13.8)	16.3	2.18–122.3	0.007
BCS	Good	334	4.79 (2.76–7.66)	Base		
	Poor	99	9.09 (4.24–16.6)	1.96	0.84–4.59	0.121
Pregnancy status	Non-pregnant	224	4.46 (2.16–8.06)	Base		0.100^a^
	First trimester	117	9.40 (4.79–16.2)	2.63	1.06–6.48	0.036
	Second trimester	57	5.26 (1.10–14.6)	1.01	0.27–3.85	0.984
	Third trimester	35	2.86 (0.07–14.9)	0.46	0.06–3.77	0.472
Milking status	Early	95	1.05 (0.03–5.73)	Base		0.026^a^
	Middle	92	15.2 (8.58–24.2)	12.2	1.52–98.1	0.019
	Late	86	10.5 (4.90–18.9)	9.29	1.14–75.7	0.037
	Dry	49	2.04 (0.05–10.9)	1.36	0.08–22.7	0.832

BCS, body condition score; CI, confidence interval; *N*, sample size. * Animals were categorized into three age groups based on the quartiles: young (1–2.5 years), adult (2.6–6 years), and old (>6 years).

a Overall *p*-value.

**Figure 2:**
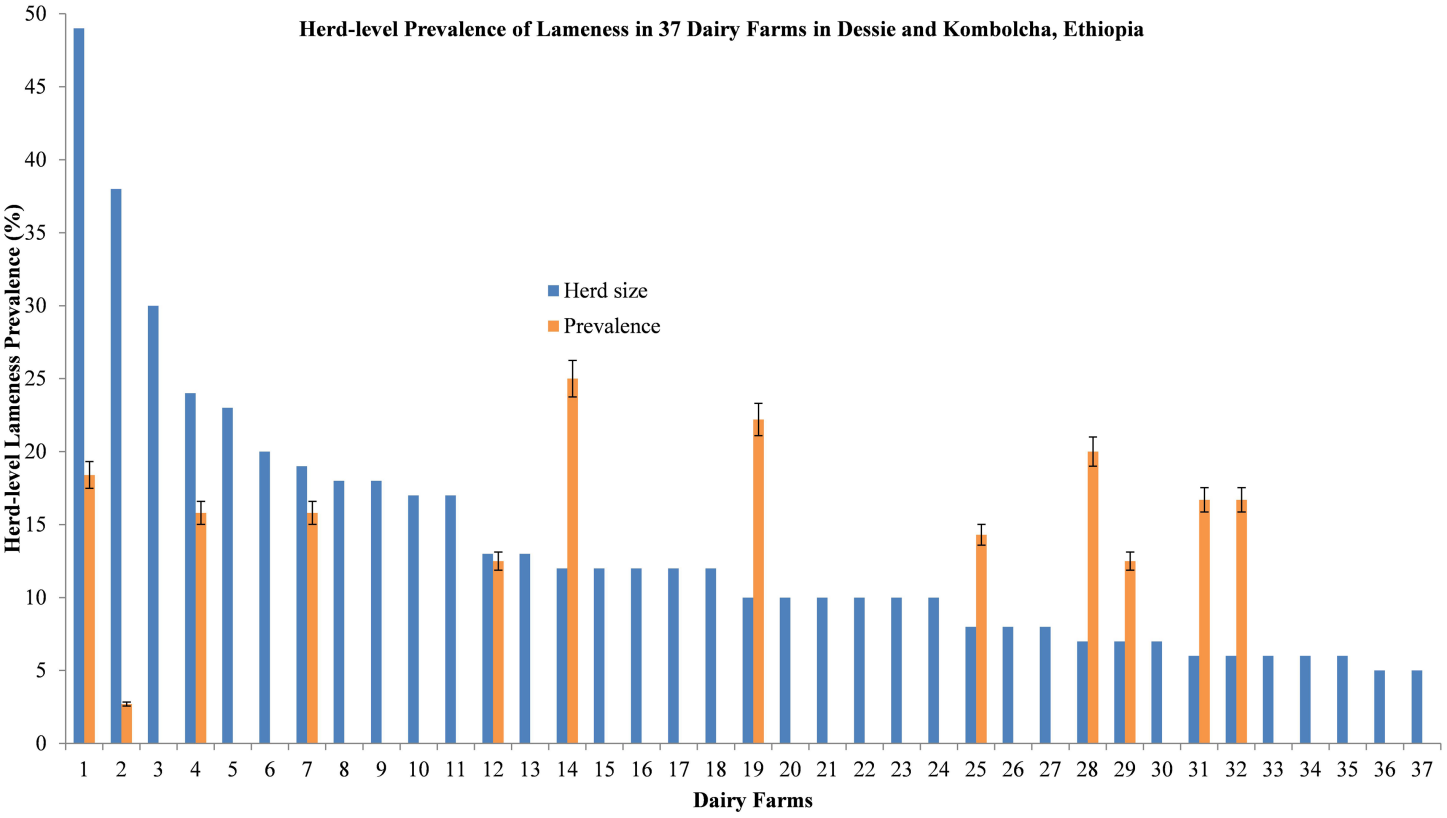
Distribution of within-herd lameness prevalence for 37 dairy herds on selected farms in Dessie and Kombolcha, South Wollo zone, Ethiopia.

The herd-level lameness prevalence was 32.4% (95% CI = 18.0–49.8%), while the average within-herd lameness prevalence was 5.20% (95% CI = 2.46–7.95%, range 0.00–25.0%), as depicted in Figure 2. The prevalence of lameness was higher in the closed house type (33.3, 95% CI = 18.0–51.8%) than in the semi-opened house type (25.0, 95% CI = 0.63–80.6%). Similarly, the prevalence was higher in the animals that were not washed (40.0, 95% CI = 5.27–85.3%) compared to those who were washed (31.2, 95% CI = 16.1–50.0%). However, the lameness prevalence was considerably higher on farms with a history of lameness (69.2, 95% CI = 38.6–90.9%) compared to those with no previous history of lameness (12.5, 95% CI = 2.66–32.4%), as shown in Table 3.

**Table 3:** Prevalence of lameness and mixed logistic regression analysis of univariable associations expressed as odds ratios for herd-level factors in 433 cows across 37 South Wollo dairy herds in Dessie and Kombolcha.

Factors	Categories	*N*	Prevalence (95%CI)	Odds ratio	95% CI	*p*-value
Herd size*	Small	19	31.6 (12.6–56.6)	Base		
	Medium	13	23.1 (5.04–53.8)	0.69	0.17–2.78	0.603
	Large	5	60.0 (14.7–94.7)	2.41	0.58–10.0	0.224
House type	Closed	33	33.3 (18.0–51.8)	Base		
	Semi-open	4	25.0 (0.63–80.6)	0.71	0.09–5.54	0.743
Barn type	Stall barn	25	24.0 (9.36–45.1)	Base		
	Loosen barn	12	50.0 (21.1–78.9)	2.53	0.80–7.93	0.112
Exercise area	No	21	33.3 (14.6–57.0)	Base		
	Yes	16	31.2 (11.0–58.7)	0.92	0.29–2.93	0.893
Animal washing	No	5	40.0 (5.27–85.3)	Base		
	Yes	32	31.2 (16.1–50.0)	0.73	0.16–3.40	0.692
Cleaning frequency	Once	2	50.0 (1.26–98.7)	Base		
	Twice	8	37.5 (8.52–75.5)	0.68	0.07–6.78	0.741
	Three times	27	29.6 (13.8–50.2)	0.51	0.06–4.21	0.529
Hoof trimming	No	25	28.0 (12.1–49.4)	Base		
	Yes	12	41.7 (15.2–72.3)	1.64	0.52–5.22	0.401
History of lameness	No	24	12.5 (2.66–32.4)	Base		
	Yes	13	69.2 (38.6–90.9)	8.83	2.34–33.3	0.001

CI, confidence interval; *N*, sample size. * Herd size was classified into three categories based on the number of animals: small (5–10), medium (11–24), and large (>24).

### 3.2 Associated animal- and herd-level risk factors of lameness

In the present study, four variables were retained in the final multivariable logistic regression model to assess animal- and herd-level risk factors at the end of the modeling process. The animal-level risk factors included in the final model were age, BCS, and milking status, while lameness history was included as a herd-level risk factor. Consequently, the odds of being lame were higher in the cows in middle lactation (OR = 10.8, 95%CI = 1.37–84.8, *p* = 0.024) and late lactation stages (OR = 11.1, 95%CI = 1.38–88.8, *p* = 0.024), as shown in Table 4. Although age was included in the final multivariable model to control for confounding effects, this factor was not associated with increased odds of a cow being clinically lame, as none of the age categories showed statistically significant *p*-values. Moreover, the cows’ body condition scores were not significantly associated with the odds of being clinically lame in the final animal- and herd-level multivariable model. However, the farms with a history of lameness (OR = 10.0, 95% CI = 2.87–37.4, *p* = 0.001) were more likely to be positive for lameness (Table 4).

**Table 4:** Multivariable mixed logistic regression analysis of animal- and herd-level risk factors for lameness in 433 dairy cows and heifers across 37 farms located in Dessie and Kombolcha, Northeast Ethiopia, between May 2022 and February 2023.

Factors	Categories	Estimate (*β*)	SE	Odds ratio	95% CI	*p*-value
Intercept		−7.65	1.57			
Age	1–2.5 years	Base				0.012^a^
	2.6 – 6 years	0.68	1.12	1.96	0.22–17.5	0.545
	>6 years	1.97	1.05	7.15	0.91–56.3	0.062
BCS	Good	Base				
	Poor	0.84	0.45	2.32	0.97–5.54	0.059
Milking status	Early	Base				0.019^a^
	Middle	2.38	1.05	10.8	1.37–84.8	0.024
	Late	2.40	1.06	11.1	1.38–88.8	0.024
	Dry	0.08	1.43	1.08	0.07–17.8	0.957
Lameness history	No	Base				
	Yes	2.34	0.66	10.0	2.87–37.4	0.001

a Overall *p*-value.

BCS, body condition score; *β*, regression coefficient; SE, standard error of *β*; CI, confidence interval of the odds ratio.

The AIC of the final full model for risk factors at animal and herd levels was lower than that of the null model, indicating a satisfactory fit. In addition, the final multivariable model for the animal- and herd-level risk factors achieved a model performance score of 60% compared to the null model. The outputs of the model performance check or diagnostics, including model-predicted intervals, uniformity of residuals, collinearity, and normality of random effects for factors at the animal and herd levels, are provided in Supplementary material 2.

## 4 Discussion

The overall animal-level prevalence of lameness found in this study was 5.8%. This result is almost comparable to the findings of a previous study (44), which reported a lameness prevalence rate of 7.7% in dairy cattle under urban and peri-urban production systems in the Addis Ababa milk shed. It also agrees with the results of another study (45), which reported a lameness prevalence rate of 7% in Danish dairy cows. However, the results of the present study are lower than those reported in other regions: 8.98% in Sululta (46), 9.2% in Debre Birhan (34), 9.7–19.2% in Hawassa, Wondo Genet, Arsi Negele, Yirga Alem, and Wolaita Sodo (26), 25.6% in intensive and extensive dairy farms of Adama (47), and 13.9% in Bishoftu (25).

On the other hand, the prevalence of lameness found in this study is higher than the rates reported in other regions: 2.94% in Mekelle (48), 3.0% in Addis Ababa (49), and 3.5% in Hawassa (27). These discrepancies in the prevalence of dairy cattle lameness across the various studies conducted in different regions (states) of Ethiopia may be due to variations in host-related factors such as breed, age, body condition status, parity, and productivity of the animals. Other likely reasons may be associated with differences in agroecology, study season, production systems, herd size, and management factors such as housing and hoof trimming (25). According to Vermunt and Malmo (50), the prevalence of lameness and the associated lesions vary widely between different management systems, such as between intensively fed cattle and cattle maintained indoors for an extended period of the year.

The prevalence of lameness in the current study is lower than that reported in other countries. This could be due to differences in management systems. In Ethiopia, particularly in the current study area, dairy farms are small-scale with less intensive management systems compared to countries such as England and Wales, where there are more intensive management systems and large-size production systems (51). The breed of cattle used for milk production in Ethiopia consists of crossbreeds (Holstein Friesian and local Zebu cattle), which may possess greater resilience to lameness than the pure-dairy breeds used in other countries. Moreover, according to a previous study (52), a higher prevalence of lameness is associated with higher milk yield. Kebede (53) reported that the average milk yield of crossbred dairy cattle in Ethiopia is 8.4 L per day, which is lower than that of many other developed countries. This might have contributed to the lower prevalence of lameness in the present study. Seasonal variations in the prevalence of lameness, along with differences in nutritional, climate, and housing conditions and methods employed for lameness detection, could also be possible reasons for the lower lameness prevalence in the present study.

According to the literature, older cows are more likely to become lame than younger ones (52, 54, 55). This may be due to extended exposure to risk factors, such as standing on hard surfaces, which can cause trauma and claw wear (56). Furthermore, hoof injuries and other musculoskeletal disorders are more difficult for older cows to recover from. This is because their tissue’s capacity to repair decreases as they age. However, age was not associated with increased odds of a cow being clinically lame in the present study. According to the current study, lameness was more common in cows with poorer body conditions, although it was not significantly associated with the odds of being clinically lame, as the *p*-values were borderline. Similarly, previous research has consistently shown that cows are at a high risk of becoming lame when their body condition score deteriorates (57, 58). A loss of body condition contributes to the pathophysiology of hoof horn lesions that cause lameness by thinning the digital cushion and destabilizing the pedal bone (59, 60). During milking, lactating cows are often required to stand for extended periods and walk longer distances, which can worsen hoof wear and increase the risk of traumatic injuries (61, 62). Furthermore, cows may be more susceptible to nutritional deficits and resultant hoof problems such as sole ulcers and white-line disease because of the metabolic demands of lactation (63, 64). Previous studies (61, 63, 65) have indicated that a poor body condition score is associated with increased odds of lameness. One possible reason for the augmenting risk of lameness in cows with a poor BCS is thin digital cushions, which can lead to claw horn disease (63). Cows with a low BCS and multiparous cows have higher odds of lameness (64). In the present study, the likelihood of lameness prevalence was strongly associated with a history of lameness on the farm (i.e., farms that reported lameness was a problem). This finding is consistent with that of a previous study (14), which found a higher prevalence of lameness in dairy farms that reported lameness as a challenge or had previously experienced lameness. The majority of farmers may overlook the extent of the problem because of the small proportion of severely lame animals on their farms, which is often underestimated. This may result in fewer endeavors and resources dedicated to controlling the problem, allowing lameness to persist on the farm.

## 5 Conclusion

According to the current study’s findings, the prevalence of lameness was low in the dairy cows under investigation. On farms where lameness had previously occurred, the prevalence of lameness was strongly correlated with cows’ middle and late lactation status. Therefore, farmers should routinely evaluate and maintain their cows’ lactation phases and increase their awareness to prevent and control lameness, thus reducing the discomfort and financial losses associated with its occurrence.

## Data Availability

The original contributions presented in the study are included in the article/Supplementary material, further inquiries can be directed to the corresponding author.

## Supplementary material

Supplementary Material 1 Questionnaire template used to collect information related to animal and herd-level risk factors for dairy cattle lameness on selected farms in Dessie and Kombolcha Town of South Wollo Zone.

Supplementary Material 2 Model performance checks and diagnostics for animal and herd-level risk factors of lameness on 37 dairy herds in Dessie and Kombolcha, Ethiopia.
