# Supplementary material for: Prevalence and risk factors for lameness in dairy cattle on selected farms located in Dessie and Kombolcha, Northeast Ethiopia
Source: Front Vet Sci. 2025 Apr 28;12:1456527. doi: 10.3389/fvets.2025.1456527 (PMC12067794; doi:10.3389/fvets.2025.1456527)
Supplement: Supplementary Material 1 — Questionnaire template used to collect information related to animal and herd-level risk factors for dairy cattle lameness on selected farms in Dessie and Kombolcha Town of South Wollo Zone. [file Data_Sheet_1.DOCX]

Supplementary material: Questionnaire for dairy cattle lameness data collection

**Wollo University**

**School Of Veterinary Medicine**

**Questionnaire interview on lameness of dairy cattle in and Dessie city and Kombolcha Town of South Wollo Zone.**

**N.B.** Please check the choices for the answer that is most accurate for your farm. For questions that ask for specific information, please give your best estimate or recollection. The questionnaire should be completed by the person who makes most decisions on the farm.

Date of interview____________________________________________________________

Farm name__________________________________________________________________

Phone number____________________________ Address____________________________

1. Management system of farm: A. intensive B. semi-intensive

2. Herd composition and size

| **Animal class** | Local | Cross | Total |
| --- | --- | --- | --- |
| Cow |  |  |  |
| Heifers |  |  |  |
| Lactating cows |  |  |  |
| Dry cows |  |  |  |
| Bull |  |  |  |
| Total herd size |  |  |  |

3. Housing and management practices

3.1. House type

A. Semi-opened B. Closed

3.2. Barn type

A. Stall bran B. Loosen barn

3.3. Floor type

A. Concrete B. Soil

3.4. House cleaning frequency per day

A. Once B. Twice C. Three times

3.5. Bedding material use:

A. Yes B. No

4. Cow scraping practice:

A. No B. Yes

5. Hoof trimming practice:

A. No B. Yes

6. Do you have exercise area and allow cow to exercises?

A. No B. Yes

7. Is the lameness problem of your farm?

A. Yes B. No

8. What measures do you taken to prevent lameness?

……………………………………………………………………………………………………………………………………………………………………………………………………………………………………………………………………………………………………………………………………………………………………………………………..

………………………………Thank You! ................................................................
