# Supplementary material for: Prevalence and risk factors for lameness in dairy cattle on selected farms located in Dessie and Kombolcha, Northeast Ethiopia
Source: Front Vet Sci. 2025 Apr 28;12:1456527. doi: 10.3389/fvets.2025.1456527 (PMC12067794; doi:10.3389/fvets.2025.1456527)

**Supplementary Materials 2: Final multivariable mixed logistic regression model for animal and herd-level risk factors.**

| ###Multivariable mixed logistic regression model  > #using 10 initial points for adaptive quadrature estimation to improve the approximations of the model  > summary(Animalclass_fm7<-glmer(VLS.2 ~ factor(AgeG) + factor(BCSed) +  + factor(Milkingstatus) + factor(Lameness.problem) + (1\|Farm.name), nAGQ = 10, data= anim_level_prev,  + family = binomial("cloglog"),  + control = glmerControl(optimizer = "bobyqa")))  Generalized linear mixed model fit by maximum likelihood (Adaptive Gauss-Hermite Quadrature,  nAGQ = 10) [glmerMod]  Family: binomial ( cloglog )  Formula: VLS.2 ~ factor(AgeG) + factor(BCSed) + factor(Milkingstatus) +  factor(Lameness.problem) + (1 \| Farm.name)  Data: anim_level_prev  Control: glmerControl(optimizer = "bobyqa")  AIC BIC logLik deviance df.resid  138.7 172.6 -60.3 120.7 313  Scaled residuals:  Min 1Q Median 3Q Max  -1.4618 -0.2045 -0.1085 -0.0577 8.9310  Random effects:  Groups Name Variance Std.Dev.  Farm.name (Intercept) 0.2096 0.4578  Number of obs: 322, groups: Farm.name, 37  Fixed effects:  Estimate Std. Error z value Pr(>\|z\|)  (Intercept) -7.65334 1.57419 -4.862 1.16e-06 ***  factor(AgeG)2 0.67514 1.11533 0.605 0.54496  factor(AgeG)3 1.96736 1.05315 1.868 0.06175 .  factor(BCSed)2 0.83973 0.44546 1.885 0.05942 .  factor(Milkingstatus)1 2.37822 1.05242 2.260 0.02384 *  factor(Milkingstatus)2 2.40272 1.06330 2.260 0.02384 *  factor(Milkingstatus)3 0.07655 1.42886 0.054 0.95727  factor(Lameness.problem)1 2.33713 0.65503 3.568 0.00036 ***  ---  Signif. codes: 0 ‘***’ 0.001 ‘**’ 0.01 ‘*’ 0.05 ‘.’ 0.1 ‘ ’ 1  Correlation of Fixed Effects:  (Intr) f(AG)2 f(AG)3 f(BCS) fc(M)1 fc(M)2 fc(M)3  factr(AgG)2 -0.641  factr(AgG)3 -0.676 0.889  fctr(BCSd)2 -0.165 0.116 0.138  fctr(Mlkn)1 -0.611 0.032 0.019 -0.061  fctr(Mlkn)2 -0.635 0.050 0.046 -0.048 0.908  fctr(Mlkn)3 -0.435 0.013 0.007 -0.057 0.682 0.668  fctr(Lmn.)1 -0.366 0.038 0.033 0.024 -0.029 0.016 -0.044  >  > plogis(fixef(Animalclass_fm7)["(Intercept)"])*100  (Intercept)  0.04742319  > Anova(Animalclass_fm7)  Analysis of Deviance Table (Type II Wald chisquare tests)  Response: VLS.2  Chisq Df Pr(>Chisq)  factor(AgeG) 8.7814 2 0.0123918 *  factor(BCSed) 3.5535 1 0.0594200 .  factor(Milkingstatus) 9.9285 3 0.0191838 *  factor(Lameness.problem) 12.7305 1 0.0003597 ***  ---  Signif. codes: 0 ‘***’ 0.001 ‘**’ 0.01 ‘*’ 0.05 ‘.’ 0.1 ‘ ’ 1  > se <- sqrt(diag(vcov(Animalclass_fm7)))  > # table of estimates with 95% CI  > (tab7 <- cbind(Est = fixef(Animalclass_fm7), LL = fixef(Animalclass_fm7) - 1.96 * se, UL = fixef(Animalclass_fm7) + 1.96 *  + se))  Est LL UL  (Intercept) -7.65333983 -10.73874283 -4.567937  factor(AgeG)2 0.67514020 -1.51091141 2.861192  factor(AgeG)3 1.96735891 -0.09681370 4.031532  factor(BCSed)2 0.83972696 -0.03337728 1.712831  factor(Milkingstatus)1 2.37821636 0.31548222 4.440950  factor(Milkingstatus)2 2.40272104 0.31865893 4.486783  factor(Milkingstatus)3 0.07655409 -2.72401758 2.877126  factor(Lameness.problem)1 2.33712964 1.05327592 3.620983  > exp(tab7)  Est LL UL  (Intercept) 4.744569e-04 2.168819e-05 0.01037935  factor(AgeG)2 1.964308e+00 2.207087e-01 17.48234998  factor(AgeG)3 7.151763e+00 9.077251e-01 56.34714234  factor(BCSed)2 2.315735e+00 9.671736e-01 5.54463723  factor(Milkingstatus)1 1.078565e+01 1.370920e+00 84.85555772  factor(Milkingstatus)2 1.105321e+01 1.375282e+00 88.83521591  factor(Milkingstatus)3 1.079561e+00 6.561063e-02 17.76314420  factor(Lameness.problem)1 1.035148e+01 2.867028e+00 37.37430216 |
| --- |
|  |
| \| > \| \| --- \| |

#Varying intercept (Farm.name)

barplot(ranef(Animal_Herd_fm7)$Farm.name[,1],

+ main="Varying intercept by farm for lameness occurrence

+ -Animal level")

>


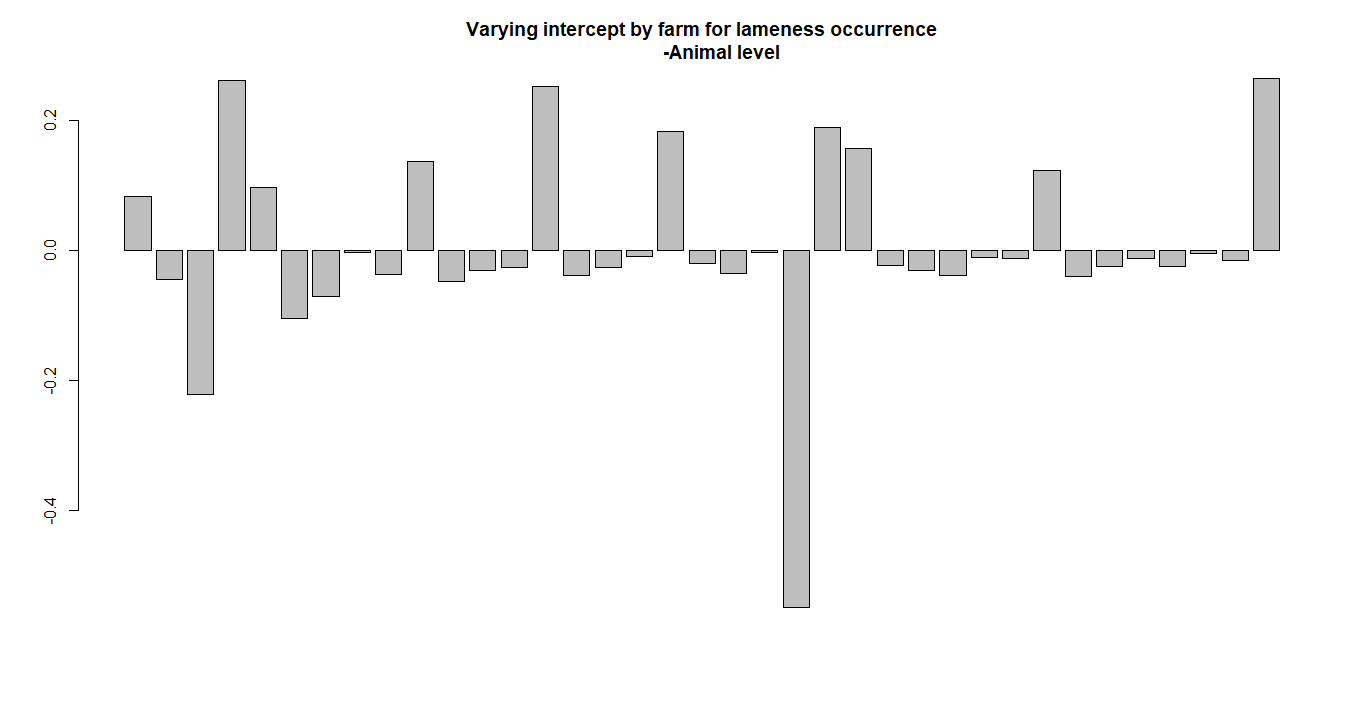


lattice::dotplot(ranef(Animal_Herd_fm7, which = "Farm.name", condVar = TRUE),

+ scales = list(y = list(alternating = 0)))


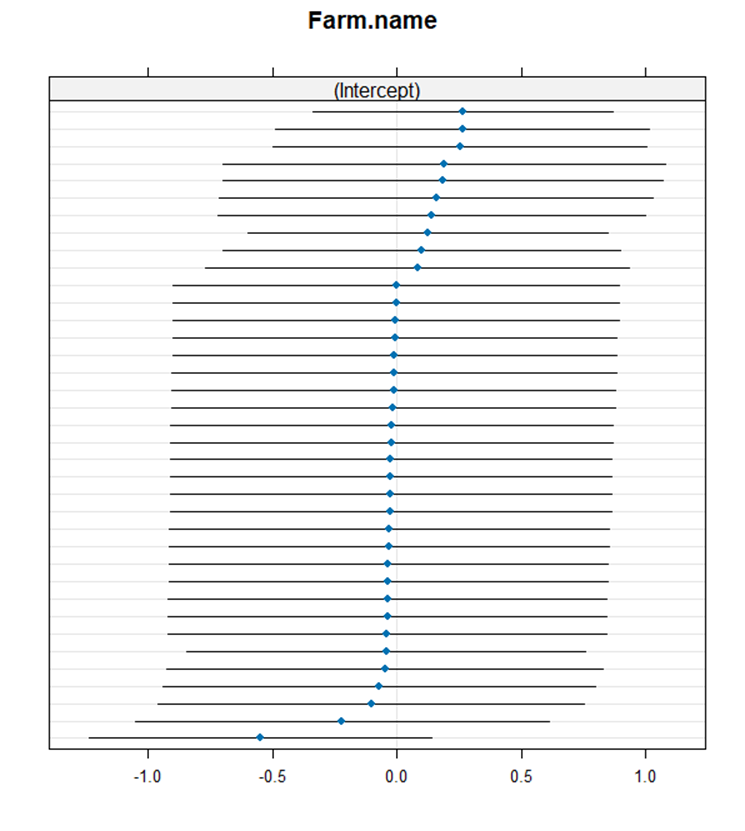


model_performance(Animal_Herd_fm7)

# Indices of model performance

AIC | AICc | BIC | R2 (cond.) | R2 (marg.) | ICC | RMSE | Sigma | Log_loss

----------------------------------------------------------------------------------------

138.675 | 139.252 | 172.646 | 0.687 | 0.647 | 0.113 | 0.224 | 1.000 | 0.176

AIC | Score_log | Score_spherical

-------------------------------------

138.675 | -2.147 | 0.046

> Anova(Animal_Herd_fm7)

Analysis of Deviance Table (Type II Wald chisquare tests)

Response: VLS.2

Chisq Df Pr(>Chisq)

factor(AgeG) 8.7814 2 0.0123918 *

factor(BCSed) 3.5535 1 0.0594200 .

factor(Milkingstatus) 9.9285 3 0.0191838 *

factor(Lameness.problem) 12.7305 1 0.0003597 ***

---

Signif. codes: 0 ‘***’ 0.001 ‘**’ 0.01 ‘*’ 0.05 ‘.’ 0.1 ‘ ’ 1

Final model diagnostics checks:

> check_model(Animal_Herd_fm7)


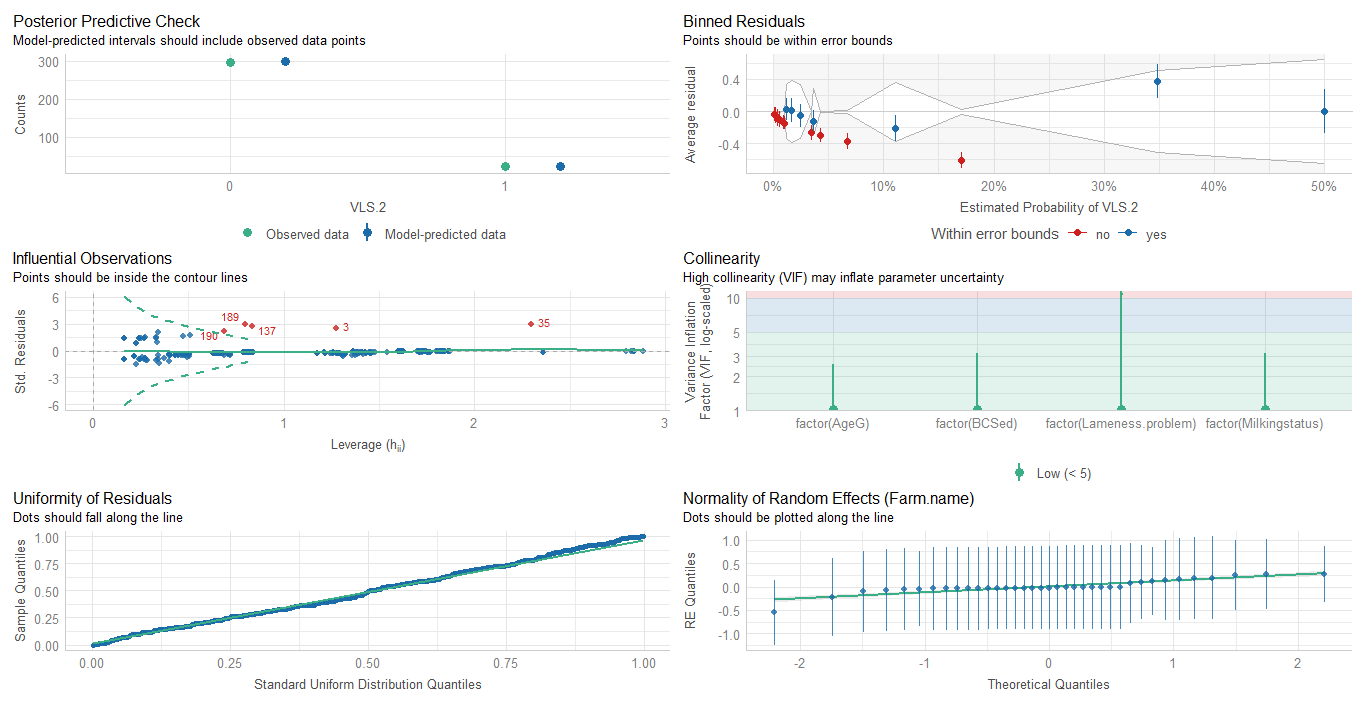


Comparison of Model Performance Indices

| Comparison of Model Performance Indices  Name \| Model \| AIC (weights) \| AICc (weights) \| BIC (weights) \| R2 (cond.)  ----------------------------------------------------------------------------------------  Null_Model \| glmerMod \| 187.6 (<.001) \| 187.7 (<.001) \| 195.8 (<.001) \| 0.410  Animal_Herd_fm7 \| glmerMod \| 138.7 (>.999) \| 139.3 (>.999) \| 172.6 (>.999) \| 0.687  Name \| R2 (marg.) \| ICC \| RMSE \| Sigma \| Log_loss \| Score_log \| Score_spherical  ---------------------------------------------------------------------------------------------  Null_Model \| 0.000 \| 0.410 \| 0.223 \| 1.000 \| 0.179 \| -1.277 \| 0.042  Animal_Herd_fm7 \| 0.647 \| 0.113 \| 0.224 \| 1.000 \| 0.176 \| -2.147 \| 0.046 |
| --- |
|  |
| \| > \| \| --- \| |

compare_performance(Null_Model, Animal_Herd_fm7,

+ rank = TRUE, verbose = FALSE)

# Comparison of Model Performance Indices

Name | Model | R2 (cond.) | R2 (marg.) | ICC | RMSE | Sigma

----------------------------------------------------------------------------

Animal_Herd_fm7 | glmerMod | 0.687 | 0.647 | 0.113 | 0.224 | 1.000

Null_Model | glmerMod | 0.410 | 0.000 | 0.410 | 0.223 | 1.000

Name | Log_loss | Score_log | Score_spherical | AIC weights

----------------------------------------------------------------------

Animal_Herd_fm7 | 0.176 | -2.147 | 0.046 | 1.000

Null_Model | 0.179 | -1.277 | 0.042 | 2.33e-11

Name | AICc weights | BIC weights | Performance-Score

----------------------------------------------------------------

Animal_Herd_fm7 | 1.000 | 1.000 | 60.00%

Null_Model | 3.07e-11 | 9.48e-06 | 40.00%

> plot(compare_performance(Null_Model, Animal_Herd_fm7,

+ rank = TRUE, verbose = FALSE))


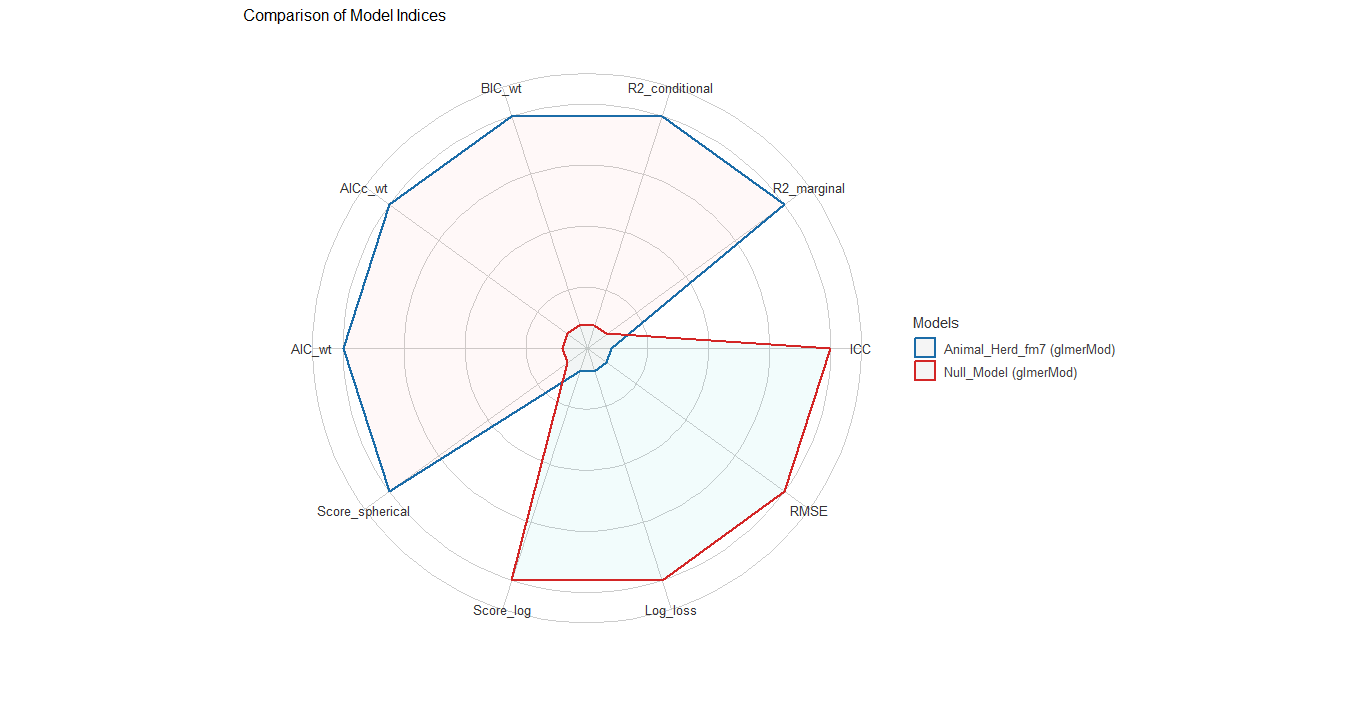

Supplement: Supplementary Material 2 — Model performance checks and diagnostics for animal and herd-level risk factors of lameness on 37 dairy herds in Dessie and Kombolcha, Ethiopia. [file Data_Sheet_2.DOCX]
